# Retinoic acid-induced upregulation of miR-219 promotes the differentiation of embryonic stem cells into neural cells

**DOI:** 10.1038/cddis.2017.336

**Published:** 2017-07-27

**Authors:** Haibo Wu, Jiamin Zhao, Beibei Fu, Songna Yin, Chao Song, Jingcheng Zhang, Shanting Zhao, Yong Zhang

**Affiliations:** 1 College of Veterinary Medicine, Northwest A&F University, 22 Xinong Road, Yangling 712100, China; 2 Key Laboratory of Animal Biotechnology, Ministry of Agriculture, Northwest A&F University, 3 Taicheng Road, Yangling 712100, China

## Abstract

MicroRNAs (miRNAs) regulate critical cell processes, such as apoptosis, proliferation, and development. However, the role of miRNAs in embryonic stem cell (ESC) neural differentiation induced by retinoic acid (RA) and factors that govern neural directional differentiation remain poorly understood. In this study, we demonstrated that miR-219 is sufficient in promoting mouse ESCs to undergo neural differentiation. We discovered that *Foxj3* and *Zbtb18*, two target genes of miR-219, are not able to determine the process of RA-induced differentiation, however they prevent ESCs from differentiating into neural cells. We identified four downstream genes, namely, *Olig1*, *Zic5*, *Erbb2*, and *Olig2*, which are essential to the gene interaction networks for neural differentiation. These data explain the mechanism of RA-induced neural differentiation of mESCs on the basis of miRNAs and support the crucial role of miR-219 in neurodevelopment.

MicroRNAs (miRNAs) are a class of non-coding, single-stranded, and 22–25-nucleotide RNA molecules that negatively regulate gene expression in plants and animals.^1^ MiRNAs mainly pair with the 3′-untranslated regions (UTRs) of a target gene and consequently degrade the target mRNA or inhibit translation. MiRNAs are involved in the regulation of embryonic development, apoptosis, and proliferation.^2, 3, 4, 5, 6, 7^ Moreover, some miRNAs, as oncogenes or tumor suppressor genes, are closely related to tumor formation.^8, 9, 10, 11^

All-trans retinoic acid (RA), a metabolic compound derived from Vitamin A, is important in cellular differentiation and neurogenesis.^12, 13, 14, 15, 16, 17^ RA is the most used morphogen for producing neural progenitor cells and neurons from pluripotent stem cells *in vitro.*^18, 19, 20, 21^ The action of RA on differentiation is probably mediated by RA receptors (RARs) in the nucleus. This action is done through the binding of the receptors to DNA sequences located in the promoter regions of RA-responsive genes.^17, 22, 23^ Furthermore, the molecular signaling pathways involved in neuronal differentiation are complex. The specific mechanism of RA-induced neural differentiation in pluripotent stem cells is not well characterized, and the role of miRNAs in this process remains unknown.^24, 25, 26^

Forkhead box J3 (*Foxj3*) belongs to a gene family of transcription factors that regulate skeletal muscle and peripheral artery developments.^27, 28^ Meanwhile, zinc-finger and BTB domain containing 18 (*Zbtb18*), also known as *Zfp238*, is involved in skeletal muscle differentiation and myogenesis.^29, 30^ When combined with PDGFR*α* and Sox6, Foxj3 and Zbtb18 can control oligodendrocyte differentiation and myelination.^31^ However, the roles of Foxj3 and Zbtb18 in stem cell differentiation are poorly understood. In this study, we demonstrated that miR-219 can promote embryonic stem cells (ESCs) to differentiate into neural cells, and *Foxj3* and *Zbtb18*, as targets of miR-219, are dominant factors in neural differentiation. These results provide a theoretical basis for RA application in ESC research and make a contribution to the understanding of neural regulatory networks.

## Results

### RA-induced miRNA expression changes in ESCs

**Figure 1:**
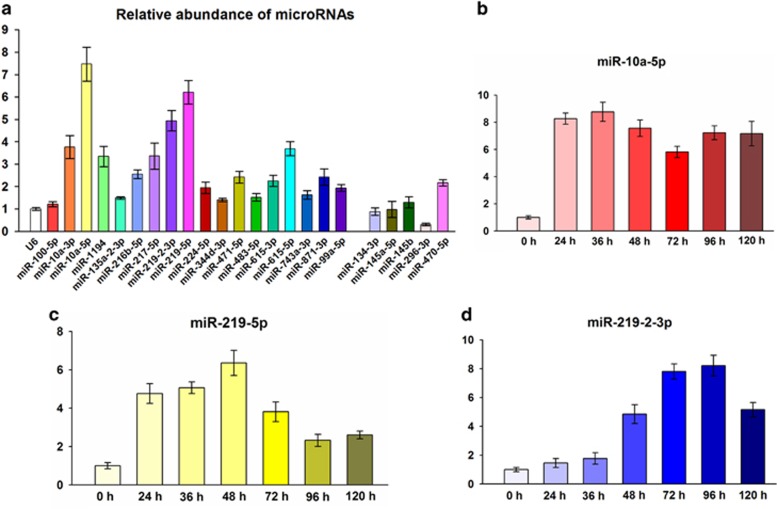
MiRNA expression changes in J1 ESCs induced by RA. (**a**) J1 ESCs were treated with RA for 48 h, and qRT-PCR was performed to determine the relative expression levels of 23 miRNAs. U6 served as the internal control. (**b–d**) Relative expression levels of miR-10-5p (**b**), miR-219 (**c**), and miR-219-2-3p (**d**) at different time points after RA treatment on ESCs. All data are presented as mean±S.D. and derived from three independent experiments

RA is widely used to induce neurogenesis.^18, 19, 20^ We determined whether or not miRNAs are involved in RA-induced neural directional differentiation by performing an array-based miRNA profiling on mouse J1 ESCs (gene expression omnibus (GEO) accession number: GSE54145; all tested miRNAs sorted by names are listed in Supplementary Data s03). The results showed that 43 miRNAs were upregulated and 281 miRNAs were downregulated after 48 h of RA treatment in ESCs. Of the 43 upregulated miRNAs (Supplementary Table S1), 18 exhibited more than a five-fold change, and five other miRNAs that are well known to be functionally related with development were then confirmed by qRT-PCR.^32, 33, 34^ Consistent with the microarray findings, the 18 changed miRNAs were upregulated by RA treatment at varying degrees(Figure 1a). Three of them, namely, miR-10a-5p, miR-219-5p, and miR-219-2-3p, showed highly significant fold changes. As previously reported, RA-induced differentiation of mESCs is powerful and irreversible, and the first 36–48 h of induction is critical.^12^ Consequently, the expression levels of these three significantly changed miRNAs were detected at different time points during RA induction (Figures 1b–d). The results showed that miR-219 was dramatically and highly expressed during the initial 48 h, and its expression decreased gradually over time (Figure 1c). This result was verified in D3 and B6 ESCs (Supplementary Figure S1A), suggesting that miR-219 plays an important role in RA-induced ESC differentiation.

### MiR-219 mediates ESCs to differentiate into neural cells

**Figure 2:**
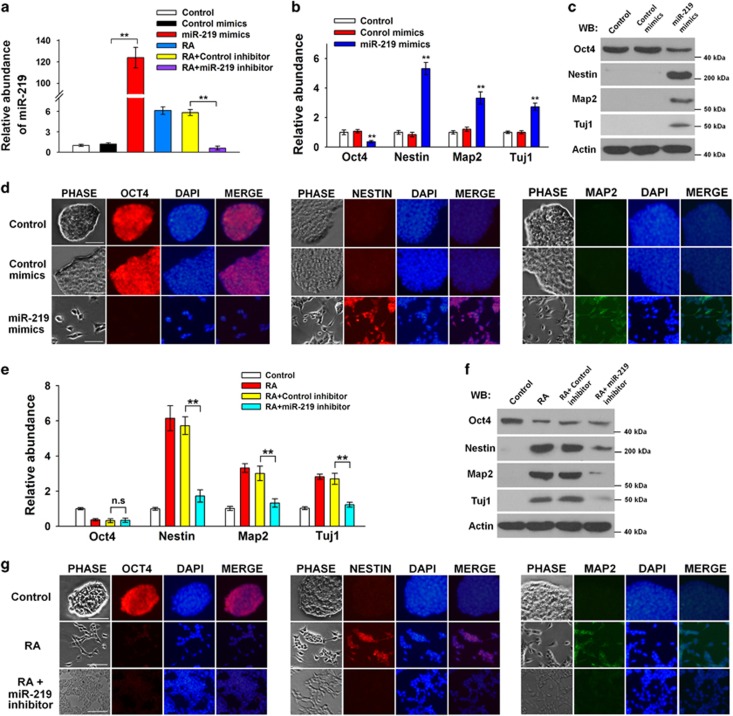
MiR-219 mediates ESCs to differentiate into neural cells. (**a**) Confirmation of the effects of miR-219 mimics and inhibitors. (**b**, **c**) ESCs were transfected with miR-219 mimics for 48 h; the relative levels of *Oct4*, *Nestin*, and *Map2* were detected through qRT-PCR (**b**) and western blot (**c**). (**d**) Immunofluorescence shows the abundance of Oct4, Nestin, and Map2, as well as the morphologies of the ESCs after transfection with miR-219 mimics for 48 h. (**e, f**) ESCs were pretreated with RA for 48 h and transfected with miR-219 inhibitors. The relative levels of *Oct4*, *Nestin*, and *Map2* were detected using qRT-PCR (**e**) and western blot (**f**). (**g**) Immunofluorescence shows the abundance of Oct4, Nestin, and Map2, as well as the morphologies of the ESCs pretreated with RA for 48 h and transfected with miR-219 inhibitors. **P<0.01; NS, no significance

Next, miR-219 mimics and inhibitors were used to investigate whether or not miR-219 is involved in RA-induced differentiation. The validity of miR-219 mimics and inhibitors were verified in Figure 2a. As expected, the mESCs differentiated into neural-type cells 48 h after transfection of miR-219 mimics. The stem cell marker *Oct4* was reduced considerably, whereas *Nestin* (a marker for neural stem cells), and *Map2* and *Tuj1* (markers for neural cells) were increased (Figures 2b–d). Furthermore, the ESCs transfected with miR-219 inhibitors resisted the RA-induced neural differentiation. As shown in Figures 2e–g, the miR-219 inhibitors blocked the RA-induced upregulation of *Nestin*, *Map2*, and *Tuj1*. The ESCs were differentiated after RA treatment regardless of the presence of miR-219 inhibitors, as characterized by the decreased Oct4 and loss of tight colony morphology (Figure 2g). Interestingly, the miR-219 inhibitors prevented ESCs from differentiating in a neural directional manner (Figures 2e–g; Supplementary Figures S1B–E). These findings suggested that miR-219 mediates ESCs to differentiate into neural cells.

### Foxj3 and Zbtb18 are the targets of miR-219

**Figure 3:**
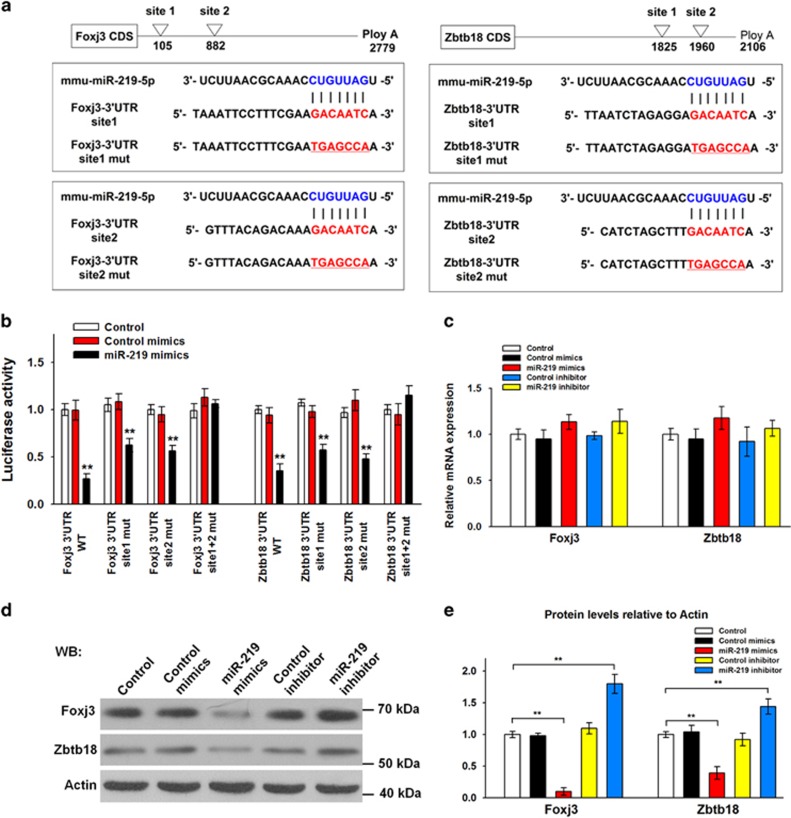
Foxj3 and Zbtb18 are the targets of miR-219**.** (**a**) 3′-UTR analysis of *Foxj3* and *Zbtb18* containing putative regions that match the seed sequence of miR-219. (**b**) At 24 h after NIH/3T3 fibroblast cells were transfected with miR-219 mimics, luciferase reporter constructs containing WT or MUT-type UTRs were transfected as indicated. Cell lysates were harvested for DLR assays. (**c**–**e**) MiR-219 mimics or inhibitors were transfected to ESCs. After 48 h, cells were harvested for qRT-PCR (**c**) and western blot (**d**) to detect the relative levels of *Foxj3* and *Zbtb18*. The relative intensities of the protein bands (**e**) were quantified with Image J software and calculated using the samples normalized to *β*-actin. All data are presented as mean±S.D. and derived from three independent experiments. Scale bars, 30 *μ*m. **P<0.01

To investigate the underlying mechanism, we examined the potential targets of miR-219 by searching the PicTar, miRanda, and targetScan databases. Among the candidate targets, the 3′-UTR of mouse *Foxj3* and *Zbtb18* contains putative regions that match the miR-219 seed sequence, which is conserved in humans and rats (Figure 3a). To confirm the predicted results, the 3′-UTRs of *Foxj3* and *Zbtb18* containing the putative regions were amplified and inserted into the psicheck-2 vector. They were then transfected to NIH/3T3 fibroblast cell line for dual luciferase reporter (DLR) assays. As shown in Figure 3b, the miR-219 mimics dramatically suppressed the activities of wild-type (WT) 3′-UTRs of *Foxj3* and *Zbtb18*. By contrast, the double mutation type (MUT) group (site 1+2 mut) with mutated seed sequences was unaffected. qRT-PCR and western blot were performed to examine the mRNA and protein levels of *Foxj3* and *Zbtb18* in ESCs transfected with miR-219 mimics or inhibitors. The results showed that the miR-219 mimics considerably decreased the protein levels of *Foxj3* and *Zbtb18* rather than the mRNA levels of these genes (Figures 3c–e). Thus, miR-219 regulates the expression levels of *Foxj3* and *Zbtb18* at the post-transcriptional level. These results indicated that *Foxj3* and *Zbtb18* are the targets of miR-219.

### Foxj3 and Zbtb18 prevent ESCs from differentiating into neural cells

**Figure 4:**
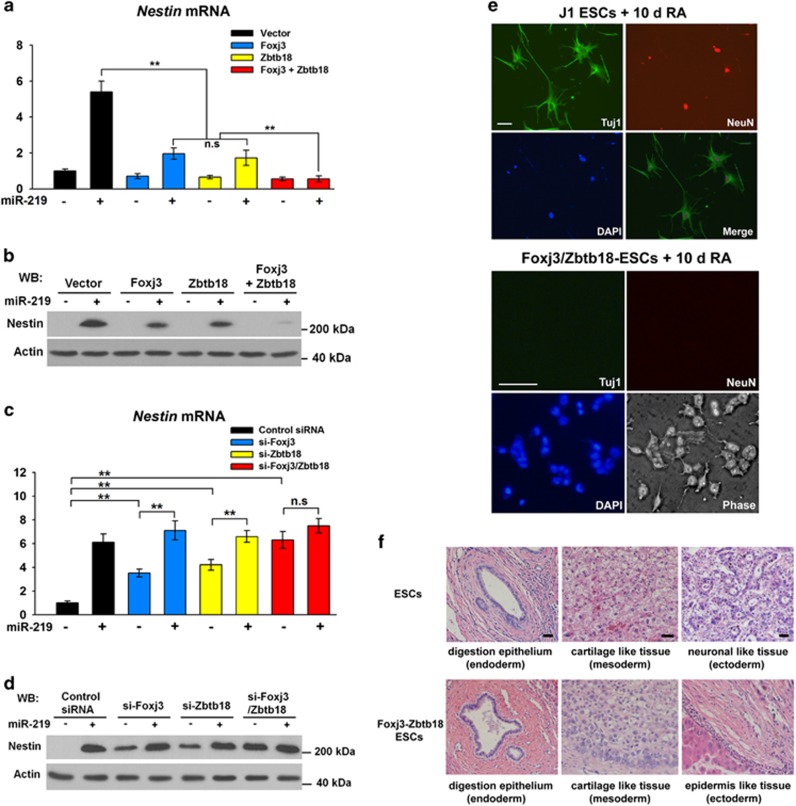
Foxj3 and Zbtb18 prevent ESCs from differentiating into neural cells**.** (**a**, **b**) ESCs were pretreated with miR-219 mimics for 24 h and followed by transfection of pCMV-Foxj3 or pCMV-Zbtb18 as indicated. After 48 h, the relative level of *Nestin* was detected through qPCR (**a**) and western blot (**b**). Actin served as the loading control. (**c**, **d**) ESCs were pretreated with miR-219 mimics for 24 h and followed by transfection of Foxj3-siRNA or Zbtb18-siRNA. After 48 h, the relative level of *Nestin* was detected through qPCR (**c**) and western blot (**d**). (**e**) Foxj3/Zbtb18-OE ESCs were induced by RA for 10 days. The morphology was observed and photographed. Scale bars: 100 *μ*m. (**f**) Control ESCs or Foxj3/Zbtb18-OE ESCs were pretreated with RA for 48 h and injected into nude mice to generate teratomas. The differentiations of the three germ layers were analyzed through H&E staining. Scale bars: 100 *μ*m. **P<0.01; NS, no significance

MiR-219 promotes the neural differentiation ESCs (Figures 2e–g), targeting *Foxj3* and *Zbtb18* (Figures 3b–d). We then investigated whether or not Foxj3 and Zbtb18 are involved in neural differentiation. Foxj3 and Zbtb18 were transiently transfected to ESCs, and the relative abundance of *Nestin* was detected. As expected, the Foxj3 or Zbtb18 disrupted the upregulation of *Nestin* after the miR-219 mimics treatment. Particularly, the synergistic effect of Foxj3 and Zbtb18 returned *Nestin* expression to basal levels (Figures 4a and b; Supplementary Figures S1F–K). Knockdown experiments were then conducted with small interfering RNA (siRNA) to verify the results. The results showed that *Nestin* expression was upregulated from 3.5- to 4.5-fold when Foxj3 or Zbtb18 was knocked down, and knockdown of both Foxj3 and Zbtb18 at one time intensified neural differentiation (Supplementary Figures 4C, D). These results suggested that Foxj3 and Zbtb18 prevent the differentiation of ESCs into neural cells. The ES cell lines that stably expressed Foxj3 and Zbtb18 were produced by pCDH-Puro-Foxj3/Zbtb18 lentivirus to investigate the functional roles of Foxj3 and Zbtb18 in neural differentiation. The resulting cell lines were used for *in vitro* differentiation under RA treatment. Compared with normal ESCs, Foxj3/Zbtb18-overexpressing (OE) ESCs differentiated but not in a neural directional manner, as characterized by morphology and the expression levels of neural markers *Tuj1* and *NeuN* (Figure 4e).

Teratoma formation tests in nude mice were performed to confirm this finding. The Foxj3/Zbtb18-OE ESCs were pretreated with RA for 48 h and injected into nude mice to generate teratomas. The teratomas were harvested 14 days later for further examination. The hematoxylin/eosin- (H&E) stained samples showed that the RA-pretreated ESCs tend to form neuronal-like tissues (Supplementary Table S2, pretreated 38.9% *versus* untreated 12.2%), and the neuronal differentiation potential of ESCs was mostly suppressed by Foxj3 and Zbtb18 (Supplementary Table S2, pretreated Foxj3/Zbtb18-ESC 5.56% *versus* pretreated ESC 38.9% representative neural-like tissues are shown in Supplementary Figure S2). Moreover, the Foxj3/Zbtb18-ESCs pretreated with RA retained the ability to form epidermis-like tissues (Figure 4f), suggesting that Foxj3 and Zbtb18 affected neuroectodermal development rather than the development of the entire ectoderm. In addition, the Foxj3/Zbtb18-OE ESCs generated less ectoderm-specific tissues (21.1% *versus* 52.2%) and more mesoderm-specific (24.4% *versus* 13.3%) and endoderm-specific (28.9% *versus* 15.6%) tissues (Supplementary Table S2) compared with RA-pretreated ESCs. We then tested the expression levels of mesoderm-specific (Brachyury, Flk1) and endoderm-specific (Sox17, Foxa2) markers in ESCs treated with RA or miR-219 antagomirs. The results showed that the increase in the expression levels of the mesoderm- and endoderm-specific markers in ESCs transfected with miR-219 antagomirs were larger than that in the RA-treated cells (Supplementary Figure S3). These data indicated that RA-induced ESCs tend to differentiate into mesodermal and endodermal cells upon miR-219 inhibition.

**Figure 5:**
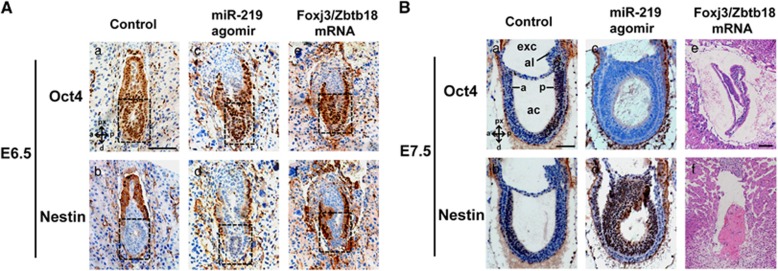
MiR-219 induces neural development in mouse embryos. MiR-219 agomirs, Foxj3/Zbtb18 mRNAs, and their corresponding negative controls were microinjected into the cytoplasm of zygotes as indicated. The expression patterns of Oct4 and Nestin in the serially sectioned E6.5 and E7.5 embryos were examined by immunohistochemistry analysis. (**A**) Immunohistochemistry analysis of the E6.5 embryos. Representative images are shown. Black dashed frames in E6.5 embryos indicate the embryonic region. (**B**) Immunohistochemistry (a–d) and H&E staining (e, f) analysis of E7.5 embryos. a and b show the representative control embryo and indicate how the embryonic region is organized and how the ectodermal and endodermal tissues can be distinguished. c and d show the representative abnormal miR-219-injected embryo, which exhibits a severe overdevelopment in its anterior region. (e, f) show representative abnormal Foxj3/Zbtb18 mRNA-injected embryo with disorganized embryonic region, apparent arrested development, or resorption. Full images of serially sectioned embryos are shown in Supplementary Figure S4. a, anterior; ac, amniotic cavity; al, allantois; d, distal; exc, exocoelomic cavity; p, posterior; px, proximal. Scale bars, 200 *μ*m

To elucidate the role of miR-219-Foxj3/Zbtb18 in the regulation of neural induction *in vivo*, we examined the effects of miR-219 and Foxj3/Zbtb18 on developing embryos between egg-cylinder and primitive streak stages. MiR-219 agomirs or Foxj3/Zbtb18 mRNAs were injected into the zygote cytoplasm. Microinjected embryos that reached the blastocyst stage were transferred to pseudopregnant female recipients. The Oct4 and Nestin expression patterns in the serially sectioned E6.5–E7.5 embryos were then examined by immunohistochemistry analysis (Figure 5). At E6.5, Nestin expression in the miR-219-injected embryos occurred earlier than the control group (Figures 5A). At E7.5, the differences between the control and miR-219-injected embryos became more apparent. Approximately 27.8% (15/54) of the miR-219-injected embryos were resorbed or had no significant progress in their development, and 38.9% (21/54) of the embryos showed varying degrees of overdevelopment in the anterior region. This finding was supported by the immunohistochemistry analysis results. In the WT embryos, Oct4 was restricted in the posterior region, and Nestin was expressed in the anterior region because of forebrain formation (Figures 5B, a and b). However, Oct4 disappeared and Nestin was detected in the entire ectoderm of the miR-219-injected embryos (Figures 5B, c and d). These results indicated that miR-219 induces neural development in the mouse embryos. On the contrast, embryos injected with Foxj3/Zbtb18 mRNAs formed egg cylinders at E6.5 (Figures 5A, e and f), but 48.8% (40/82) of the sectioned embryos showed quick degeneration and various abnormal phenotypes, 23.2% (19/82) of the embryos were resorbed completely or left a trace of residual pyknotic tissue by E7.5 (Figure 5B, e and f; Supplementary Figure S4). The extremely high rate of mutants in the miR-219- and Foxj3/Zbtb18-injected embryos implied that the normal expression of Foxj3/Zbtb18 is crucial in early embryonic development in mice.

### Olig1 and Olig2 are important in miR-219-mediated neural differentiation

The total mRNAs of Foxj3-ES and Zbtb18-ES were extracted and used for RNA-seq and transcriptome analyses to understand the specific mechanism by which Foxj3 and Zbtb18 control neural differentiation of ESCs. Compared with control, 1331 genes were differentially expressed (*P<*0.05) by two-fold in the Foxj3-ESCs (557 were underexpressed and 774 were overexpressed), and 1175 genes were differentially expressed (*P<*0.05) by two-fold in Zbtb18-ESCs (548 were underexpressed and 627 were overexpressed). Gene ontology (GO) analysis indicated that 53 GO terms in Foxj3-ES and 54 GO terms in Zbtb18-ES were enriched (*P<*0.01; Supplementary Figures S5A, B). Foxj3 and Zbtb18 work synergistically to control neural differentiation, and thus the genes located in the four overlapping biological processes (i.e., nervous system development, central nervous system development, neuron fate commitment, and forebrain development) of the enriched GO terms were selected for analysis. A total of 37 candidate genes in the Foxj3-ES group and 39 candidate genes in the Zbtb18-ES group were present. Between these groups, 17 genes overlapped, namely *Arid1a*, *Camk2b*, *Chd7*, *Erbb2*, *Glis2*, *Gnao1*, *Id2*, *Ina*, *Map1s*, *Ndn*, *Numbl*, *Olig1*, *Olig2*, *Shank1*, *Smarcd3*, *Zic2*, and *Zic5.* We hypothesized that these genes contribute to RA-mediated neural differentiation. Their expression levels were verified through qRT-PCR (Supplementary Figure S5C; Supplementary Table S3).

**Figure 6:**
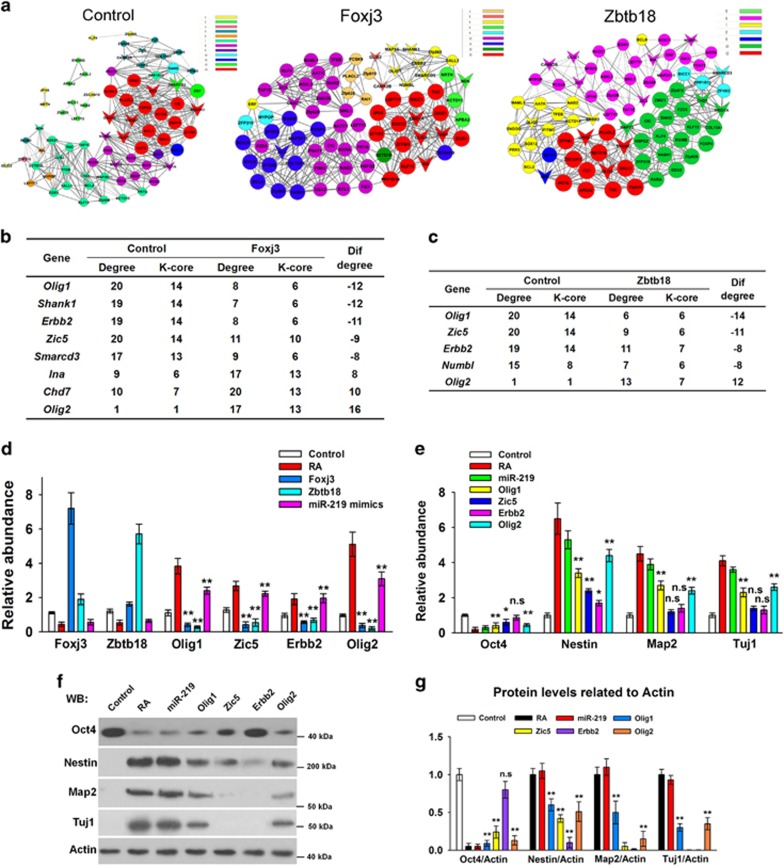
Olig1, Zic5, Erbb2, and Olig2 participate in neural regulatory networks. (**a**) Co-expression networks of differentially expressed genes in the control, Foxj3-ES, and Zbtb18-ES groups. The overlapping genes were selected to construct gene co-expression networks. Sixteen genes located in the four focused GO terms are arrow-shaped. Solid lines, positively corrected; dashed lines, negatively corrected. (**b, c**) The genes with difference degrees above eight between Foxj3-ES (**b**) or Zbtb18-ES (**c**) and control ESCs. (**d**) The relative levels of *Olig1*, *Zic5*, *Erbb2*, and *Olig2* in the ESCs were detected through qRT-PCR. The ESCs were treated or transfected with RA, pCMV-Foxj3, pCMV-Zbtb18, or miR-219 for 48 h. (**e–g**) Relative levels of *Nestin* and *Map2* were detected through qRT-PCR (**e**) and western blot (**f**) in ESCs transfected with Olig1-, Zic5-, Erbb2-, or Olig2-encoding plasmids for 48 h. Relative intensities of protein bands (**g**) were quantified by using Image J software and calculated by using the samples normalized to *β*-actin. All data are presented as mean±S.D. and derived from three independent experiments. *P<0.05; **P<0.01; NS, no significance

To locate the core regulatory genes involved in neural regulatory networks, gene co-expression networks were constructed with the overlapping genes in the Foxj3-ES and Zbtb18-ES (Figure 6a). Core regulatory factors were then identified on the basis of the degree of differences between the control and Foxj3/Zbtb18-ES groups. The genes with a difference degree of more than eight were selected. Five genes, namely, *Olig1*, *Zic5*, *Erbb2*, *Numbl*, and *Olig2*, met the specification in the Zbtb18-ES group. Meanwhile, eight genes, namely, *Olig1*, *Shank1*, *Erbb2*, *Zic5*, *Smarcd3*, *Ina*, *Chd7*, and *Olig2,* met the specifications in the Foxj3-ES group. In addition, the following genes were present in both groups: *Olig1*, *Zic5*, *Erbb2*, and *Olig2* (Figures 6b and c). We then performed qRT-PCR to validate the relationships of these genes with miR-219, Foxj3, and Zbtb18. As expected, *Olig1*, *Zic5*, *Erbb2*, and *Olig2* were upregulated two-fold to five-fold when the ESCs were induced by RA treatment or miR-219 mimics, whereas they were dramatically inhibited when Foxj3 or Zbtb18 was overexpressed (Figure 6d). These findings were consistent with the RNA-seq results. To understand the roles of Foxj3, Zbtb18, and the four genes involved in transcription factor networks during RA-dependent neural differentiation, we examined the relative expression levels of these genes in the first 10 days of RA-induced differentiation (Supplementary Figure S6). The results showed tha*t Foxj3 and Zbtb18* exhibited a similar expression pattern, that is, their expression levels rapidly declined in the first 2 days, and the temporary increased, and finally returned to a relatively low level during neural differentiation (Supplementary Figures S6A, B). However, the expression levels of *Olig1* and *Olig2* progressively increased and a sudden rise was observed around day 7 after RA-induced neural differentiation (Supplementary Figures S6C, F). This effect may be attributed to the presence of glial cells and neurons on that day.

**Figure 7:**
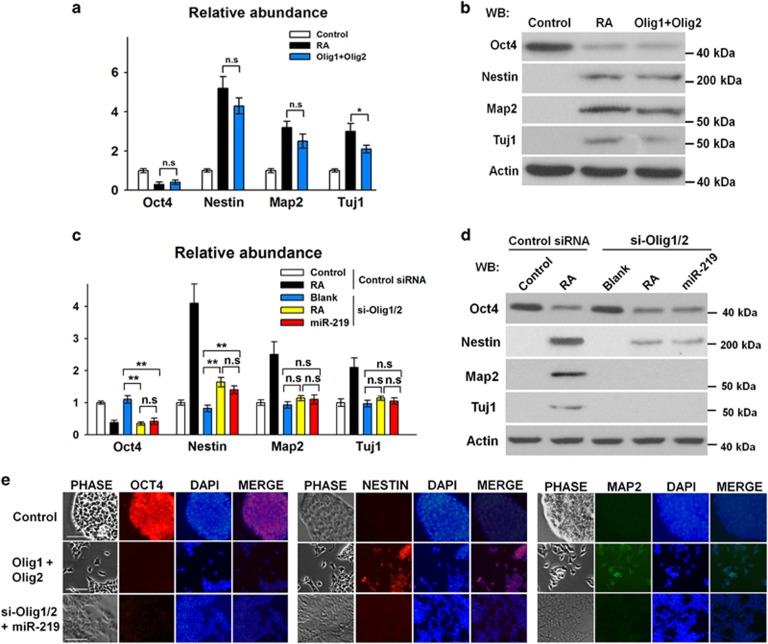
Olig1 and Olig2 are critical to miR-219-mediated neural differentiation. (**a, b**) Relative levels of *Nestin*, *Map2,* and *Tuj1* were detected through qRT-PCR (**a**) and western blot (**b**) in ESCs transfected with Olig1/Olig2-encoding plasmids for 48 h. (**c, d**) ESCs were transfected with siRNAs that were targeted to *Olig1*/*Olig2* for 12 h and treated with RA or miR-219 mimics for another 36 h. Relative levels of *Nestin*, *Map2*, and *Tuj1* were detected through qRT-PCR (**c**) and western blot. (**e**) Immunofluorescence shows the abundance of Oct4, Nestin, and Map2, as well as the morphologies of ESCs transfected with Olig1/Olig2 or miR-291 mimics. **P<0.01; NS, no significance

Furthermore, we evaluated whether these four genes are involved in the neural differentiation. As shown in Figures 6e–g, ESCs were differentiated into neural-type cells 48 h after expression of plasmids that encode Olig1, Zic5, Erbb2, or Olig2 were transfected, as characterized by the dramatic *Oct4* reduction and *Nestin* increase. These results suggested that these four factors are involved in neural regulatory networks. Notably, only Olig1 and Olig2 facilitate the expression levels of *Map2* and *Tuj1* (neural cell markers) in the ESCs (Figures 6e–g). We hypothesized that Olig1 and Olig2 are the most dominant downstream factors of miR-219-mediated neural differentiation. Olig1/2 overexpression and knockdown were performed to demonstrate the involvement of Olig1 and Olig2 in the downstream of the RA-miR-219-Foxj3/Zbtb18 pathway. The results showed that *Nestin, Map2*, and *Tuj1* dramatically increased in ESCs with overexpressed Olig1/Olig2, and the levels of neural differentiation were approximate to RA treatment (Figures 7a and b). Moreover, *Olig1/2*-knockdown ESCs cultured in the presence of RA or transfected with miR-219 mimics failed to differentiate into neural cells, as characterized by the reduction of *Oct4* and absence of significant increase in neural cell markers (*Map2* and *Tuj1*) (Figures 7c–e). These results indicated that Olig1 and Olig2 are the main factors regulated by miR-219 in RA-induced neural differentiation.

## Discussion

RA can induce various types of cells in a concentration-dependent manner. For example, a high RA concentration increases the rate of neural differentiation, whereas a low RA concentration induces cardiomyocyte differentiation of mESCS.^35^ Okada *et al.*^36^ reported that RA concentration regulates dorsoventral identity, that is, high RA concentration induces a dorsal phenotype, and low RA concentrations induces a ventral phenotype during *in vitro* mESC differentiation. A similar phenomenon was observed in human pluripotent stem cells, in which low-dose RA increased hematopoietic progenitor cell generation, whereas low-dose RA abrogated blood cell generation *in vitro.*^37^ Here, we found that 1 *μ*M RA is sufficient to induce mESCs that cultured without any stromal cell line, neural culture medium, or neural growth factors undergo neural differentiation.

During early embryonic development, RA can facilitate embryonic positioning along the embryonic axis by acting as an intercellular signaling molecule that guides the development of the posterior portion of the embryo.^18, 38^ Efforts have been undertaken to uncover the mechanism of the effects of RA in embryonic development. The most familiar mechanism is the binding of RA to RAR, which is bound to a DNA region called the RA response elements and affects the binding of other transcription factors.^39, 40, 41^ Moreover, RA acts through the *Hox* genes, which ultimately control the anterior and posterior patterns in early developmental stages.^13^ RA also affects the changes in the epigenetic marks on histones and creates a heritable change in chromatin responsiveness.^42, 43, 44^ Kashyap *et al.*^45^ reported that coincident with the RA-induced transcriptional activation of the *Hoxa* and *Hoxb* cluster genes, the epigenomic configuration of these clusters is rapidly remodeled by H3K4me3 and acH3. Notably, RA treatment stabilizes p53 in human ESCs, and p53 activates the expression levels of miR-34a and miR-145, which then repress the stem cell factors *Oct4, Klf4, Lin28a,* and *Sox2* and accelerate ESC differentiation.^46^ These findings suggested that miRNAs are important in the regulation of RA-associated differentiation of ESCs.

To this end, we characterized the expression of miRNAs in mESCs with RA treatment for 48 h. Our previous studies showed that ESCs start to differentiate irreversibly after RA pretreatment for 48 h and continued to do so even after RA withdrawal.^12^ Therefore, miRNAs with high expression levels during the early stage of RA induction may be critical in RA-induced neural directional differentiation of mESCs. We performed most of the experiments in J1 ESCs to investigate the miR-219-dependent neural regulatory networks, which was later confirmed in D3 and B6 ESCs and in C57BL/6 mice, to ensure that this finding is not a particular characteristic of a single cell line.

MiR-219 is necessary in promoting oligodendrocyte differentiation, and partially rescues oligodendrocyte differentiation defects caused by total miRNA loss.^31^ Kocerha *et al.*^47^ reported that miR-219 is important in the expression of behavioral aberrations associated with NMDA receptor hypofunction. Santa-Maria *et al.*^48^ found that miR-219 downregulation promotes neurodegeneration by targeting Tau. Recent studies have suggested that miR-219 regulates NPC proliferation and differentiation.^49, 50^ However, the specific role of miR-219 in RA-associated ESC differentiation is not elucidated. In this study, we found that miR-219 is sufficient in promoting neural differentiation by targeting *Foxj3* and *Zbtb18*. On the basis of the results of the RNA-seq performed on Foxj3-ESCs and Zbtb18-ESCs, we constructed three gene co-expression networks and identified 17 core genes that are functionally related to neurodevelopment. Interestingly, 16 of these genes were upregulated during neural differentiation, showing that most factors that dominate neural differentiation can be induced. We then demonstrated that Foxj3 and Zbtb18 are the molecular switches in the neurodevelopment system. Furthermore, we identified four critical genes by constructing gene co-expression networks. Two of them, namely, *Olig1* and *Olig2* might be the most essential elements in neural differentiation of ESCs. *Olig1*/*2*-knockdown ESCs cultured in the presence of RA or transfected with miR-219 mimics failed to differentiate into neural cells, as characterized by the absence of increase of neural cell markers (*Map2* and *Tuj1*). Notably, *Nestin* (neural stem cell marker) appeared in the *Olig1*/*2*-knockdown ESCs treated with RA or miR-219 mimics. This finding may be attributed to other factors or mechanisms involved in the miR-219-mediated neural differentiation apart from *Olig1*/*2*. However, this hypothesis requires validation through further experiments. Moreover, the mechanism of how RA upregulates miR-219 is not well understood. We speculate that histone demethylases accelerate DNA demethylation at the promoter region of miR-219, thereby increasing the expression level of miR-219 and facilitating neural differentiation. In addition to epigenetic modifications, other transcription factors can possibly mediate the effects of RA on miR-219 upregulation.^51^ Thus the involvement of histone demethylases or other transcription factors with the RA and miR-219 pathway is interesting for further investigation.

**Figure 8:**
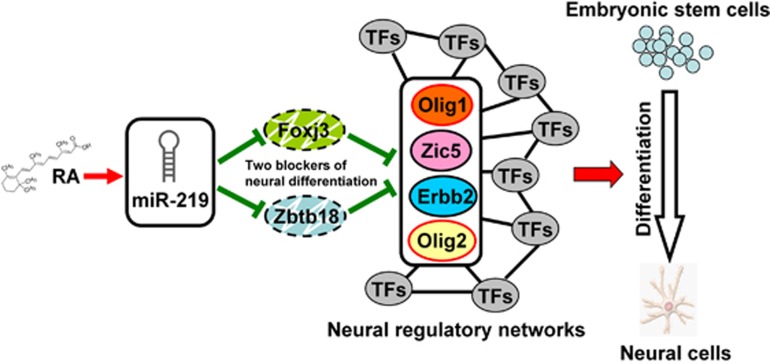
Schematic representation of RA-induced neural differentiation of ESCs. RA treatment led to upregulation of miR-219, which targeted Foxj3 and Zbtb18. Downregulation of Foxj3 and Zbtb18 resulted in the activation of Olig1, Zic5, Erbb2, and Olig2. The neural regulatory networks were triggered, and the neural directional differentiation of ESCs was determined. TF, transcription factor

Overall, we discovered that functionally important gene expression changes mediated by miRNA contribute to RA-induced differentiation. We demonstrated that *Foxj3* and *Zbtb18*, two target genes of miR-219, are the main controls in neural differentiation of ESCs (Figure 8). Our findings illustrate the mechanism of miRNA-mediated neural differentiation of ESCs, as well as the plasticity and dynamic nature of the gene regulatory networks during neural differentiation. However, the Foxj3 and Zbtb18 regulatory activities dependent on other transcription factors remain unclear. The specific mechanism of Foxj3 and Zbtb18 for determining the fate of neural differentiation and the functional roles of Foxj3 and Zbtb18 in multiple biological processes requires further investigation.

## Materials and methods

### Ethic statement

This study was carried out in strict accordance with the Guidelines for the Care and Use of Animals of Northwest A&F University. All animal experimental procedures were approved by the Animal Care Commission of the College of Veterinary Medicine, Northwest A&F University. C57BL/6 mice were purchased from Xi’an Jiao-tong University, China. Five-week-old female athymic nude mice were purchased from The Fourth Military Medical University. Every effort was made to minimize animal pain, suffering, and distress, and reduce the number of animals used.

### Cell culture, transient transfection, and treatment

J1, R1, and D3 mESCs were purchased from American Type Culture Collection (ATCC, Manassas, VA, USA), and grown adherent to plastic plates (Corning Costar, Cambridge, MA, USA) coated with matrigel (Geltrex, Thermo Fisher Scientific, San Jose, CA, USA) and maintained under a feeder-free and serum-free system. The ESC culture medium was composed of Knockout Dulbecco's modified Eagle's medium (Knockout DMEM, Thermo Fisher Scientific), 15% (v/v) Knockout Serum Replacement (Thermo Fisher Scientific), 1 × non-essential amino acids (Thermo Fisher Scientific), 100 *μ*M *β*-mercaptoethanol (Millipore, Bedford, MA, USA), 2 mM glutamine (Thermo Fisher Scientific), 50 units/ml penicillin, 50 *μ*g/ml streptomycin, and 1000 units/ml LIF (ESGRO, Millipore). The growth condition of mESCs was consistent throughout this study.

Plasmids were transfected with Xfect Transfection Reagent (Clontech, Palo Alto, CA, USA) following the manufacturer’s instructions. The transfection efficiency was monitored by a backbone vector with an extra eGFP element and calculated by flow cytometry. The average transfection efficiency was ~75%–85%, and the representative result is shown in Supplementary Figure S7. Double-stranded mmu-miR-219-5p (miRBase accession number MIMAT0000664, 5'-UGAUUGUCCAAACGCAAUUCU-3') mimics, single-stranded mmu-miR-219-5p inhibitors and their corresponding negative controls were purchased from GenePharma (GenePharma, Shanghai, China). Negative control siRNA and siRNAs that target *Foxj3*, *Zbtb18*, *Olig1*, and *Olig2* were chemically synthesized by Ribobio (Guangzhou, Guangdong, China). The knockdown efficiencies of siRNAs were verified by qRT-PCR and western blot (Supplementary Figure S8). The miRNAs and siRNAs were transfected with Lipofectamine RNAiMAX reagent (Invitrogen, Life Technologies, Carlsbad, CA, USA) following the manufacturer’s instructions. RA (all-*trans*-Retinoic acid, product number: R2625) was purchased from Sigma-Aldrich (St. Louis, MO, USA). A stock solution of 1 mM RA was prepared in absolute ethanol, stored at −20 °C, and protected from light. The working concentration of RA was 1 *μ*M. Culture medium and RA were changed daily and ESCs were passaged every 2–3 days at a 1 : 3–1 : 5 ratio according to cell density. A direct induction system without any stromal cell line, neural culture medium, or neural growth factors as neural inducer was performed to render the normal cultured ESCs a perfect control.

### Construction of plasmids

Full-length coding sequences of *Foxj3* (NCBI accession number NM_172699) and *Zbtb18* (NCBI accession number NM_001012330) were amplified from J1 cDNA. These fragments were inserted into pCMV-HA (Clontech; verified in Supplementary Figure S8), pCDH-MCS-T2A-Puro-MSCV (System Biosciences, Palo Alto, CA, USA), and pCAG-EGFP (kindly provided by Dr Wei Zhang) vectors. The 3′-UTR of *Foxj3* (1084 bp in length. Located at bases 2041–3124 refer to NM_172699.3; –34 to +1050 if consider base 2075 as the start site of UTR) and *Zbtb18* (936 bp in length. Located at bases 2886–3821 refer to NM_001012330.1; +1141 to + 2076 if consider base 1746 as the start site of UTR) were amplified from J1 cDNA and inserted into the psicheck-2 vector (Promega, Madison, WI, USA) through standard molecular cloning methods and confirmed by sequencing. All the primers used for plasmid construction are listed in Supplementary Tables S4 and S5.

### Luciferase assays

Luciferase assays were performed using the DLR Assay System (Promega) as previously described.^52^ In brief, ESCs were cotransfected with a luciferase reporter construct and internal control plasmid pRL-SV40 (Promega). Cells were lysed 24 h after transfection or treatment, and relative luciferase activities were measured by firefly luciferase luminescence divided by Renilla luciferase luminescence.

### Real-time qRT-PCR

We used qRT-PCR to analyze miRNA expression. Total RNA samples (including small RNA molecules) were isolated using mirVana miRNA Isolation Kit (Ambion, Austin, TX, USA). Purified RNA was reverse-transcribed using a miScript II RT Kit (Qiagen, Hilden, Germany). The expression of mature miRNAs was quantified using a miScript SYBR Green PCR Kit, which contained 10 × miScript Universal Primer (Qiagen), and was performed according to the manufacturer’s instructions. Quantization of U6 was performed to normalize miRNA expression levels.

We also used qRT-PCR to analyze gene expression. Total RNA samples were isolated using Trizol reagent (Invitrogen). Purified RNA was reverse-transcribed using a SYBR PrimeScript RT–PCR Kit (Takara, Otsu, Shiga, Japan). The expression of mRNAs was quantified using a SYBR Premix ExTaq II Kit (Takara). Real-time PCR was performed on an ABI StepOnePlus PCR system (Applied Biosystems, Foster City, CA, USA), and results were normalized to *β*-actin mRNA levels. Data were analyzed using the 2^−ΔΔCt^ method. Primer sequences used for qPCR are listed in Supplementary Tables S6 and S7.

### Western blot analysis

Western blot analysis was performed as previously described.^52^ Blots were probed with 1/1000 rabbit anti-Oct4 (#2788), 1/1000 rabbit anti-MAP2 (#4542) (Cell Signaling Technology, Beverly, MA, USA), 1/500 rabbit anti-Foxj3 (#SAB2100844), 1/500 rabbit anti-Zbtb18 (#SAB2103436), 1/1000 mouse anti-Nestin (#MAB5326), 1/1000 rabbit anti-Brachyury (#B8436), 1/2000 mouse anti-*β*-actin (#A5441) (Sigma-Aldrich), 1/1000 goat anti-Flk1 (VEGF receptor 2, #AF644; R&D Systems, Minneapolis, MN, USA), 1/1000 mouse anti-Sox17 (#ab192453), 1/1000 rabbit anti-Foxa2 (#ab108422) (Abcam, Cambridge, MA, USA), and 1/1000 mouse anti-Tuj1 (neuronal class III *β*-tubulin, #MMS-435P; Covance, Princeton, NJ, USA). Immunoblots were revealed by autograph using SuperSignal west pico substrate (Thermo Fisher Scientific). The relative intensity of the protein bands was quantified using Image J software (NIH, Bethesda, MD, USA) and calculated by samples normalized to the controls. All data were presented as mean±S.D. and derived from three independent experiments.

### RNA-seq and data analysis

Total RNA was extracted from Foxj3-ESCs or Zbtb18-ESCs by Trizol reagent (Invitrogen) separately. Then, the RNA samples were sent to Novel Bioinformatics company (Shanghai, China) for RNA-seq. The RNA quality was checked by Bioanalyzer 2200 (Agilent Technologies, Santa Clara, CA, USA) and kept at −80 °C. The RNA with RIN>8.0 is right for cDNA library construction. RNA libraries were prepared for sequencing using IonProton. The cDNA libraries were processed for the proton sequencing process according to the commercially available protocols. Data were submitted to the GEO archive. The accession number is GSE61748. All tested genes sorted by fold changes are listed in Supplementary Data s04-s06. Pathway analysis was used to find out the significant pathway of the differential genes according to KEGG database. Fisher’s exact test was calculated to select the significant pathway, and the threshold of significance was defined by *P*-value and false discovery rate (FDR).^53^

GO analysis was performed to facilitate elucidating the biological implications of unique genes in the significant or representative profiles of the differentially expressed gene in the experiment. GO annotations were downloaded from NCBI (http://www.ncbi.nlm.nih.gov/), UniProt (http://www.uniprot.org/), and the GO (http://www.geneontology.org/). Fisher’s exact test was applied to identify the significant GO categories and FDR was used to correct the *P*-values.

Gene co-expression networks were presented to find the relations among genes. Gene co-expression networks were built according to the normalized expression values of genes selected from genes in significant GO terms. For each pair of genes, we calculate the Pearson correlation and choose the significant correlation pairs (FDR<0.05) to constructed the network.^54^ Degree centrality is defined as the link numbers one node has to the other.^55^ While considering genes in different networks, core regulatory factors were determined by the degree differences (Dif degree) between control ESCs and Foxj3/Zbtb18-ES. K-cores were also introduced to simplify the graph topology analysis. K-core means that at least K other genes are connected to a node.^56, 57^

### Teratoma formation, H&E staining, and scoring method

The ESCs with stable expressions of Foxj3/Zbtb18 were pretreated with RA for 48 h, and injected to nude mice to generate teratomas. The differentiations of the three germ layers were analyzed by H&E staining. In brief, teratomas were fixed with 10% buffered formaldehyde for more than 24 h, embedded in paraffin, sectioned, and stained with H&E according to the standard procedure. To evaluate the potential of three germ layer differentiation, we generated nine teratomas from the control ESCs, RA-pretreated ESCs, and RA-pretreated Foxj3/Zbtb18-OE ESCs, respectively. We obtained 10 slices from each teratoma, and each slice was observed by three operators. Individual germ layer-specific tissue scores were added to calculate the germ layer score. The scores of the nine teratomas were added to determine the percentages of three germ layer differentiations of the control ESCs, RA-pretreated ESCs, or RA-pretreated Foxj3/Zbtb18-OE ESCs. The scoring of all the slices was performed by the same operators for consistency.

### Embryo preparation, microinjection, and immunohistochemistry analysis

For embryo preparation, C57BL/6 mice were maintained under controlled lighting conditions (12 h light : 12 h dark). After mating, the morning when the vaginal plug appeared was designated as embryonic day 0.5 (E0.5). Mouse zygotes were collected and denuded of cumulus cells. MiR-219 agomir, antagomir, and their corresponding negative controls were chemically synthesized (Ribobio) and diluted with a dilution buffer (Ribobio) to a final concentration of 5 nM. The Foxj3/Zbtb18 mRNAs were synthesized *in vitro* using the T7 Quick Coupled Transcription/Translation System (Promega) and adjusted to a final concentration of 50 ng/*μ*l according to the manufacturer’s instructions. Approximately 10 pl of miR-219 agomirs or Foxj3/Zbtb18 mRNAs were injected into the cytoplasm of the zygotes under a Zeiss Axio Observer Z1 fluorescence microscope (Carl Zeiss, Jena, Germany) equipped with an Eppendorf Transfer-Man NK2 micromanipulator (Eppendorf, Hamburg, Germany). The microinjected embryos were cultured in M16 (Sigma-Aldrich) medium at 37 °C at a humidified atmosphere containing 5% CO_2_ to enable their development to the blastocyst stage.

Pseudopregnant female mice (recipients) were mated with vasectomized males 3.5 days prior to embryo transfer. Well-developed blastocysts with similar morphologies were then selected for embryo transfer in each group. Twelve blastocysts were transferred to each recipient, with six embryos in each uterine horn.

Recipients were killed 3 or 4 days after embryo transplantation to recover E6.5 or E7.5 embryos. The embryos were dissected in DMEM with 10% FBS. The conceptuses covered with decidual mass were extracted from the uterus and fixed in 4% paraformaldehyde. The embryos were then processed for paraffin wax embedding. Then, sections with thickness of 5 *μ*m were obtained, dewaxed in xylene, and rehydrated through an ethanol series into PBS. The sections were incubated with mouse anti-Nestin (1 : 100, #MAB5326; Sigma-Aldrich) or rabbit anti-Oct4 antibody (1 : 100, #SAB2701972; Sigma-Aldrich) at 4 °C overnight, and then incubated with horseradish peroxidase-conjugated secondary antibody at room temperature for 2 h. Photographs were obtained with fluorescent microscope (Carl Zeiss).

### Statistical analysis

All data are presented as mean±S.D. and derived from at least three independent experiments. Statistical significances were analyzed using the Student’s *t-*test. A value of *P<*0.05 was considered significant.

### Data availability

All data in this paper have been deposited to the GEO of NCBI. The accession numbers are GSE54145 and GSE61748.
