# Supplementary material for: Retinoic acid-induced upregulation of miR-219 promotes the differentiation of embryonic stem cells into neural cells
Source: Cell Death Dis. 2017 Jul 27;8(7):e2953–. doi: 10.1038/cddis.2017.336 (PMC5550877; doi:10.1038/cddis.2017.336)
Supplement: Supplementary Materials [file cddis2017336x1.pdf]

Supplementary Materials

Title: Retinoic acid-induced upregulation of miR-219 promotes the differentiation of embryonic stem cells into neural cells

Authors: Haibo Wu[1, 2, *], Jiamin Zhao[1, 2], Beibei Fu[1, 2], Songna Yin[2], Chao Song[1, 2], Jingcheng Zhang[1, 2], Shanting Zhao[1] and Yong Zhang[1, 2, *]

[1]College of Veterinary Medicine, Northwest A&F University, Yangling 712100, Shaanxi, China

[2]Key Laboratory of Animal Biotechnology, Ministry of Agriculture, Northwest A&F University, Yangling 712100, Shaanxi, China

[*]Corresponding author

Contact: hbwu029@nwsuaf.edu.cn (H.W) or zhangy1956@sina.com (Y.Z)

Running title: MiR-219 promotes neural differentiation

Keywords: Differentiation; Embryonic stem cells; MicroRNA; Co-expression network

Contain: Supplementary Figures S1-S8; Supplementary Tables S1-S7

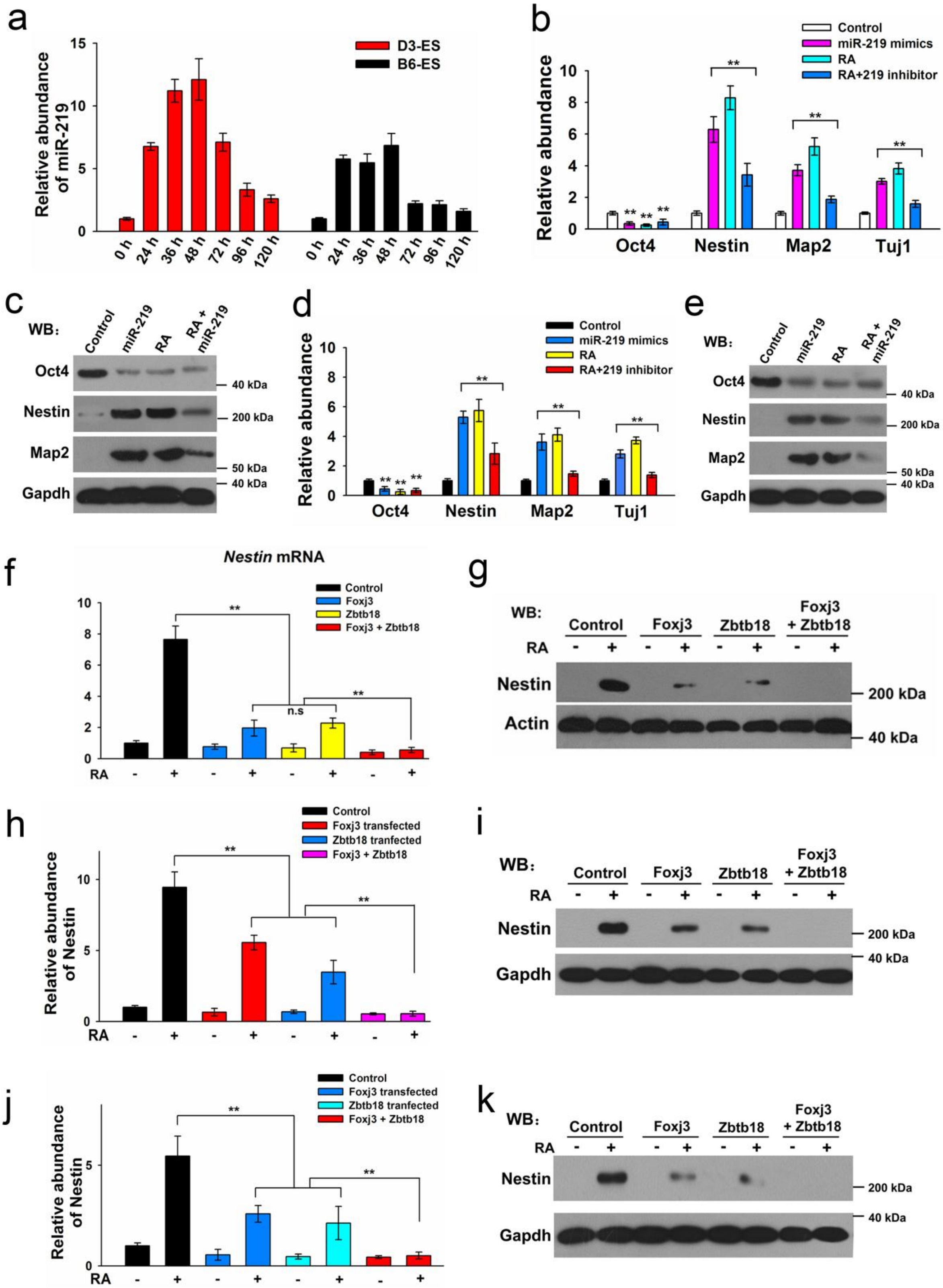

Fig. S1 MiR-219-mediated neural differentiation is conserved in D3 and B6 mESCs. (A): Relative expression levels of miR-219 at different time points after RA treatment in D3 and B6 mESCs. (B–E): D3 ESCs (B, C) or B6 ESCs (D, E) were treated with RA and transfected with miR-219 mimics or miR-219 inhibitors for 48 h.

Relative levels of *Oct4, Nestin, Map2,* and *Tuj1* were detected through qRT-PCR (B, D) and Western blot (C, E). (F–K): J1 ESCs (F, G), D3 ESCs (H, I) or B6 ESCs (J, K) were pretreated with RA for 24 h and transfected with pCMV- Foxj3 or pCMV-Zbtb18. Relative level of *Nestin* was detected through qPCR (F, H, J) and Western blot (G, I, K). Gapdh served as the loading control. **: $P < 0.01$.

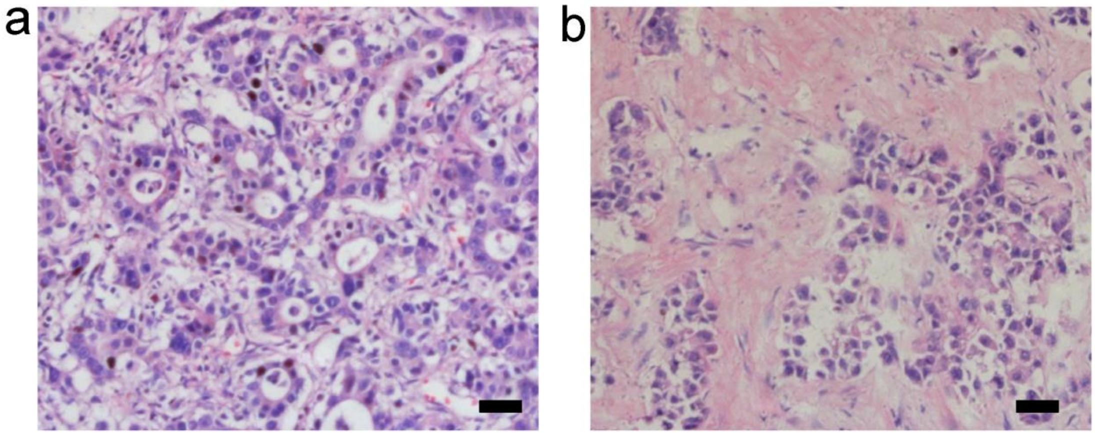

Fig. S2 Representative neural like tissues derived from RA pretreated ESCs (a) and RA pretreated Foxj3/Zbtb18-ESCs (b). Scale bars: 100 μm.

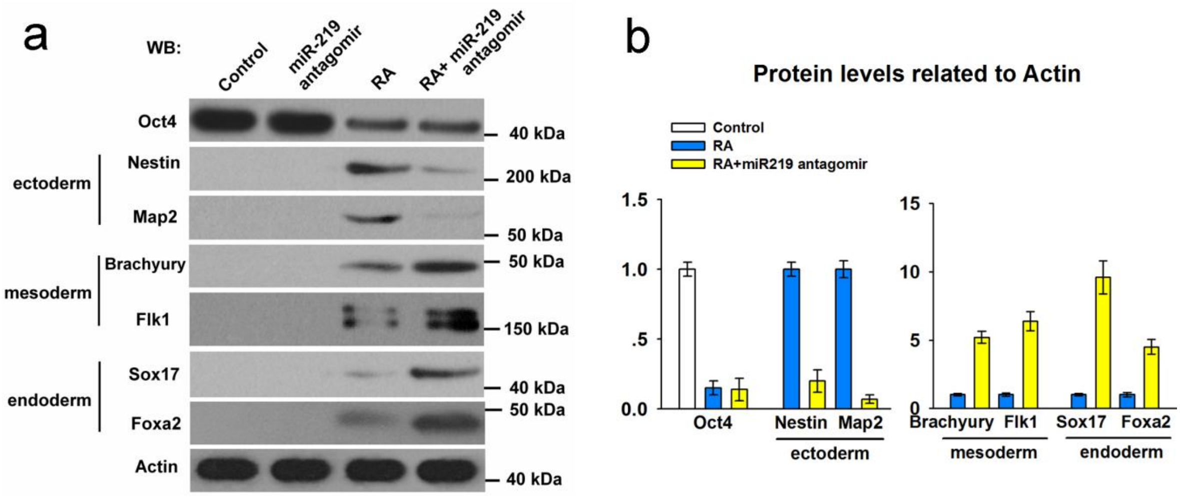

Fig. S3 RA-induced ESCs tend to differentiate into mesodermal and endodermal cells upon miR-219 inhibition. (A): ESCs were treated with RA or miR-219 antagomirs for 48 h. Western blot was performed to detect the expression levels of ectoderm- (Nestin, Map2), mesoderm- (Brachyury, Flk1), and endoderm- (Sox17, Foxa2) specific markers. (B): Relative intensities of the protein bands were quantified with Image J software and calculated by using the samples normalized to β-actin. All data are presented as mean ± SD and derived from three independent experiments.

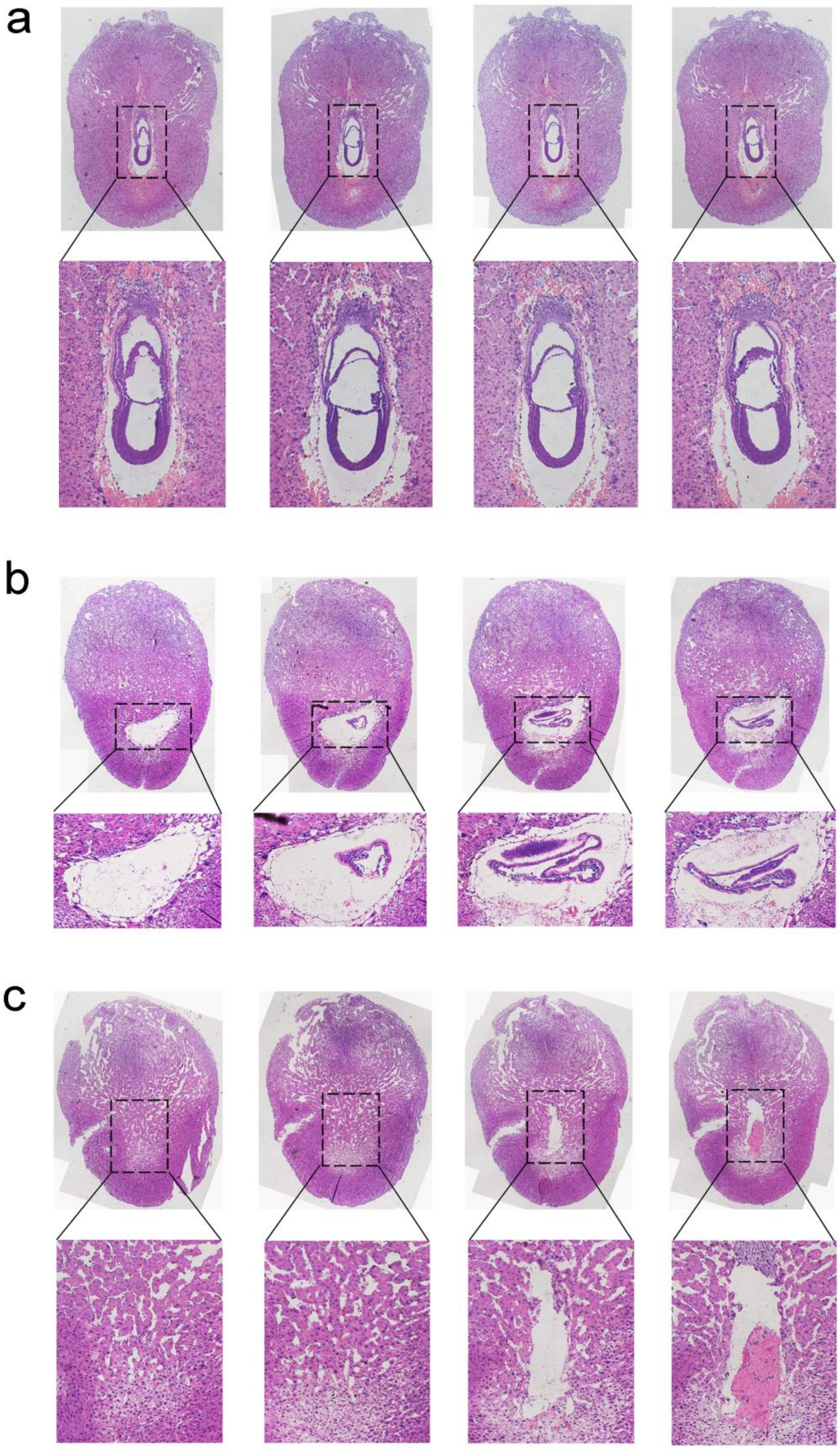

Fig. S4 Representative images of serially sectioned abnormal embryos. (A): Normal E7.5 embryos injected with control mRNA transcribed from empty vectors. (B, C): Representative abnormal E7.5 embryos injected with *Foxj3*/*Zbtb18* mRNAs. (B) shows the quick degeneration and displays an abnormal phenotype of the embryonic zone. (C) shows apparent resorption and a trace of residual pyknotic tissue.

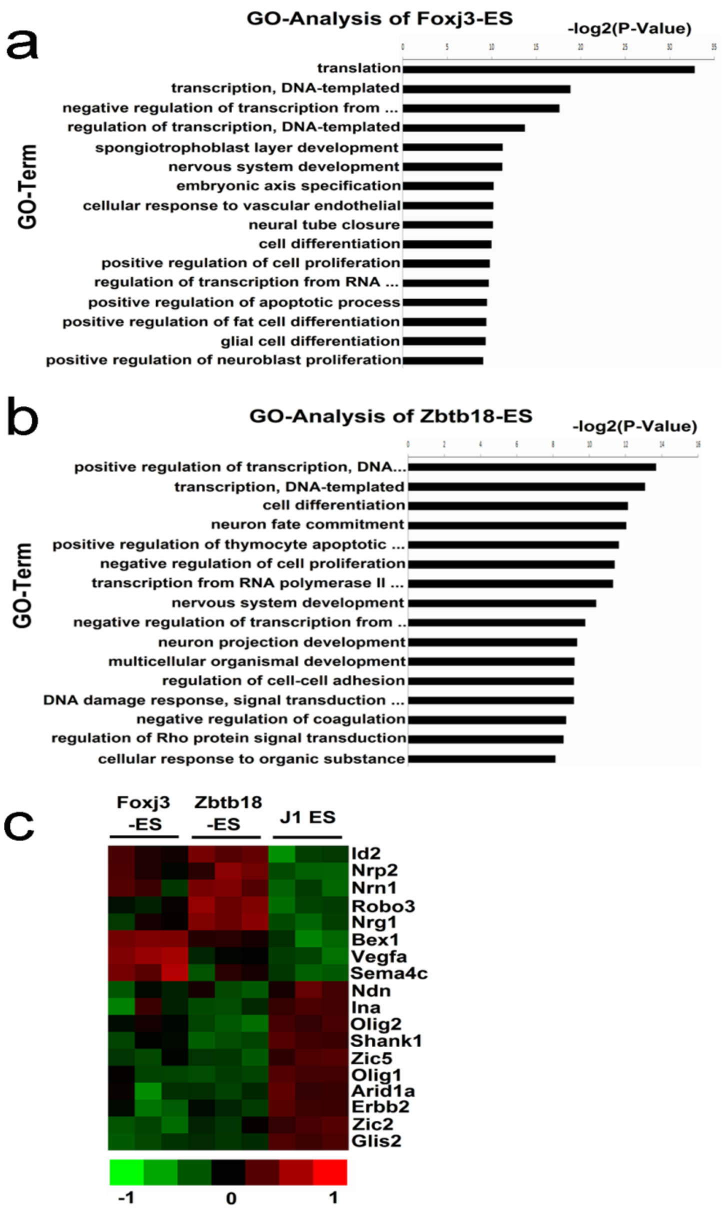

Fig. S5 Top-ranking GO terms and involved genes. (A, B): Top-ranking GO terms are listed according to *P* values. Five GO terms, involved in nervous system development, embryonic axis specification, neural tube closure, glial cell differentiation, and positive regulation of neuroblast proliferation, were significantly enriched ($P < 0.05$) in Foxj3-ESCs (A). Similarly, four GO terms, involved in neuron fate commitment, nervous system development, neuron projection development, and regulation of Rho protein signal transduction, were significantly enriched ($P < 0.05$) in Zbtb18-ESCs (B). (C): Heat map representation of the selected genes involved in nervous system development, central nervous system development, neuron fate commitment, and forebrain development. Gene expression is shown with pseudocolor scale (−1 to 1).

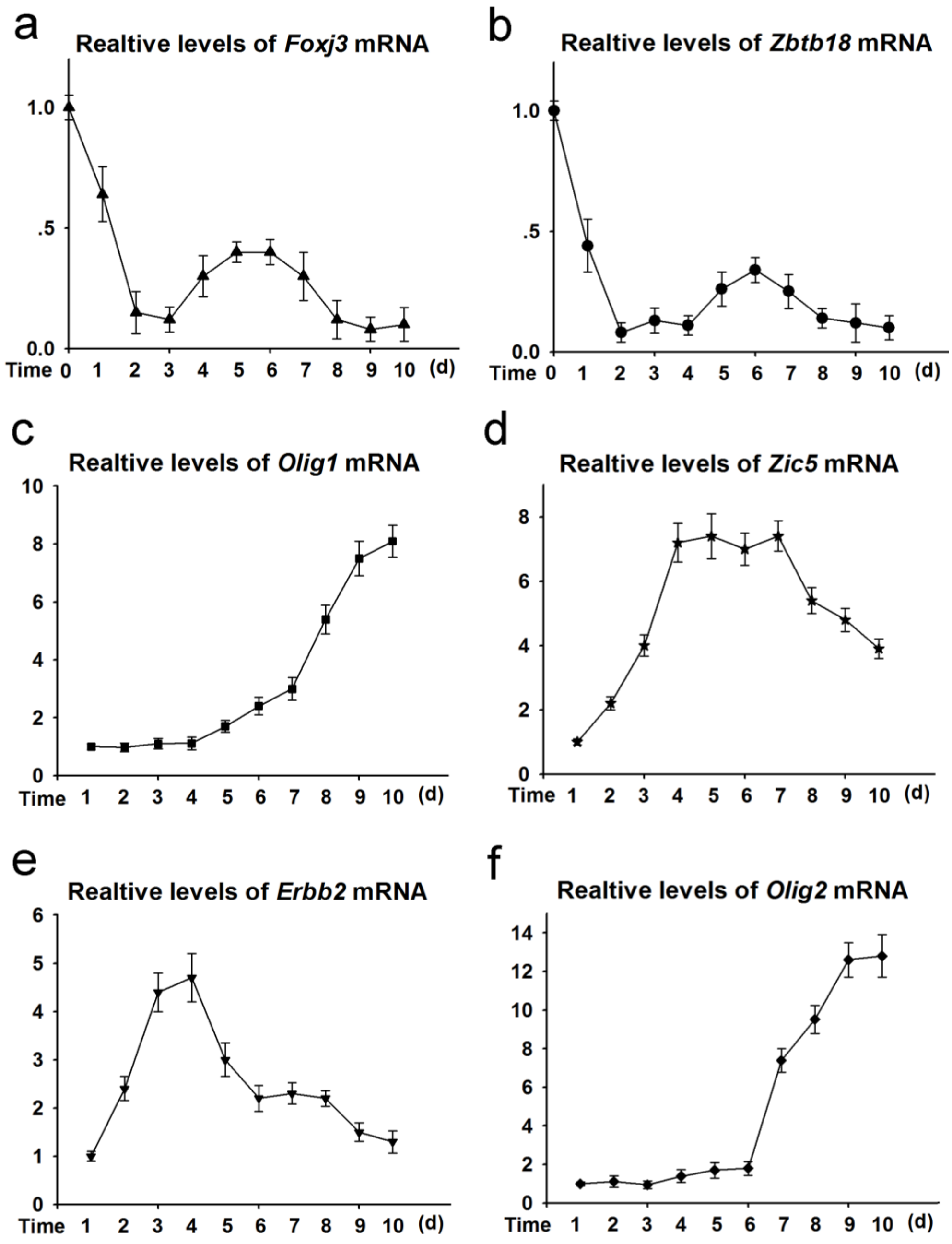

Fig. S6 Expression patterns of *Foxj3*, *Zbtb18*, *Olig1*, *Zic5*, *Erbb2*, and *Olig2* during RA-induced neural differentiation. mESCs were normally cultured and induced by 1 μM RA for 10 d. Culture medium and RA were changed daily. Relative expressions of *Foxj3* (A), *Zbtb18* (B), *Olig1* (C), *Zic5* (D), *Erbb2* (E), and *Olig2* (F) during RA-induced neural differentiation were examined through qRT-PCR.

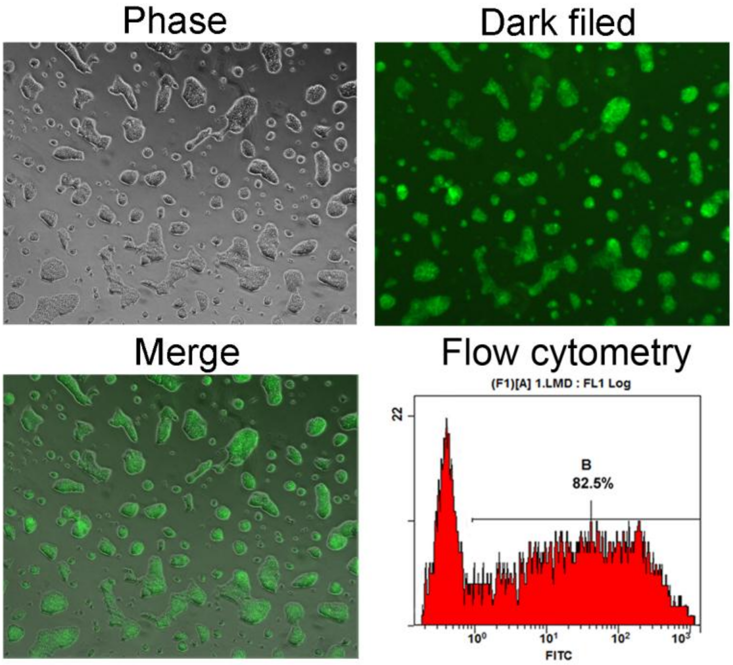

Fig. S7 Representative result of the efficiency of plasmid transfection.

Transfection efficiency was monitored by a backbone vector with an extra eGFP element and calculated through flow cytometry. Fluorescent photograph and flow cytometry analysis of ESCs transfected with eGFP vectors.

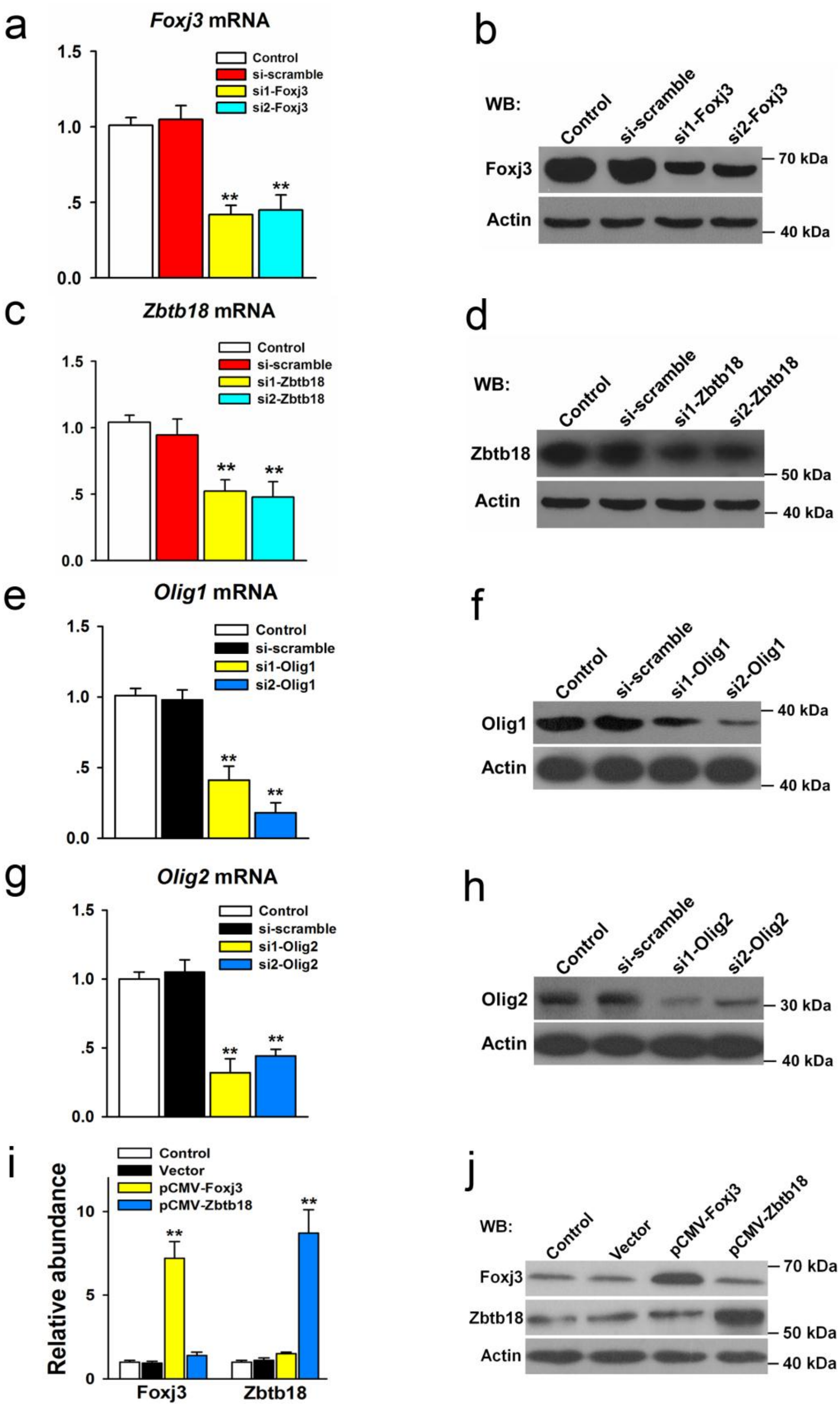

Fig. S8 Validation of siRNAs and vectors. (A-H): ESCs were transfected with *Foxj3*, *Zbtb18*, *Olig1*, or *Olig2* siRNAs for 48 h. Cell lysates were harvested, and qPCR and Western blot were performed to verify the efficiency of *Foxj3* (A, B), *Zbtb18* (C, D), *Olig1* (E, F), or *Olig2* (G, H) knockdowns. (I, J): Validation of pCMV-Foxj3 and pCMV-Zbtb18 plasmids by qPCR (I) and Western blot (J).

Table S1 **Microarray data of 43 upregulated miRNAs**

miR-name	fold-change	miR-name	fold-change
mmu-miR-100-5p	5.19	mmu-miR-465a-3p	2.89
mmu-miR-10a-3p	49.99	mmu-miR-465a-5p	2.19
mmu-miR-10a-5p	38.94	mmu-miR-465b-3p	2.89
mmu-miR-10b-5p	3.83	mmu-miR-465c-3p	2.88
mmu-miR-1194	6.82	mmu-miR-470-5p	2.62
mmu-miR-135a-2-3p	14.12	mmu-miR-471-5p	6.17
mmu-miR-138-5p	3.54	mmu-miR-483-5p	5.58
mmu-miR-149-5p	2.82	mmu-miR-500-3p	2.57
mmu-miR-181a-5p	3.31	mmu-miR-615-3p	118.72
mmu-miR-181b-5p	2.79	mmu-miR-615-5p	314.87
mmu-miR-188-5p	2.92	mmu-miR-669f-3p	2.23
mmu-miR-216b-5p	154.85	mmu-miR-741-3p	3.09
mmu-miR-217-5p	1285.28	mmu-miR-741-5p	4.29
mmu-miR-219-2-3p	15.88	mmu-miR-743a-3p	5.11
mmu-miR-219-5p	6.89	mmu-miR-743b-3p	3.57
mmu-miR-224-5p	5.36	mmu-miR-871-3p	7.99
mmu-miR-335-3p	2.06	mmu-miR-878-5p	2.39
mmu-miR-335-5p	2.41	mmu-miR-881-3p	3.28
mmu-miR-344-3p	2.09	mmu-miR-883a-3p	2.57
mmu-miR-344d-3p	10.23	mmu-miR-883b-5p	4.51
mmu-miR-375-3p	2.26	mmu-miR-99a-5p	27.03
mmu-miR-452-5p	2.09		

Table S2 **Potential of three germ layer differentiation**

Germ layer	mESC	RA pretreated for 48 h	
		mESC	Foxj3-Zbtb18 mESC
Neuronal like tissue positive	**11(12.2%)**	**35(38.9%)**	**5(5.56%)**
Ectoderm	**29(32.2%)**	**47(52.2%)**	**19(21.1%)**
Mesoderm	**23(25.6%)**	**12(13.3%)**	**22(24.4%)**
Endoderm	**25(27.8%)**	**14(15.6%)**	**26(28.9%)**

Table S3 **Verification of gene expression changes by qRT-PCR**

Gene ID	Fold Change			Sequence of primers used in q-PCR
	Foxj3-ES	Zbtb18-ES	q-PCR of miR-219 treated ES	
Arid1a	0.39	0.36	2.42	F:CTTCCCCAACCACCAGTACAA<br>R:CTGTGCGAAGGACGAAGAC
Camk2b	0.35	0.44	1.74	F:CGTTTCACCGACGAGTACCAG<br>R:GCGTACAATGTTGGAATGCTTC
Chd7	0.44	0.42	3.07	F:GTGAAGCTGTGTTGAAAGGCA<br>R:CTCGGCAAAGCTCCTCTTCTG
Erbb2	0.46	0.48	2.14	F:ACCGACATGAAGTTGCGACTC<br>R:AGGTAAGCTCCAAATTGCCCT
Glis2	0.30	0.31	4.26	F:GACGAGCCCCTCGACCTAA<br>R:AGCTCTCGATGCAAAGCATGA
Gnao1	0.26	0.15	7.20	F:TGCACGAGTCTCTCATGCTCT<br>R:AGATGGTCAAGGGTGACTTCT
Id2	2.16	2.97	0.34	F:ATGAAAGCCTTCAGTCCGGTG<br>R:AGCAGACTCATCGGGTCGT
Ina	0.49	0.37	3.51	F:GCGCAGTATGAGTCCCTGG<br>R:CGGTACTCGTGGATCTCCTCT
Map1s	0.37	0.33	2.67	F:GAGTTGGAACGAGGTGTTCG<br>R:TCACAATGCTGGAGAAGGTG
Ndn	0.45	0.39	1.43	F:GAGGTCCCCGACTGTGAGAT<br>R:TGCAGGATTTTAGGGTCAACATC
Numbl	0.46	0.42	1.94	F:GCAGGCACCATGAACAAGTTA<br>R:TCTTCACAAACGTGCATTCCC
Olig1	0.38	0.30	3.87	F:TCTTCCACCGCATCCCTTCT<br>R:CCGAGTAGGGTAGGATAACTTC
Olig2	0.44	0.23	5.61	F:TCCCCAGAACCCGATGATCTT<br>R:CGTGGACGAGGACACAGTC
Shank1	0.39	0.26	3.37	F:TGCATCAGACGAAATGCCTAC<br>R:AACAGTCCATAGTTCAGCACG
Smarcd3	0.39	0.23	3.16	F:CCCGAGTCCCAGGCTTACA<br>R:GCTTTCGCTTTTGCTTCATGG
Zic2	0.33	0.42	2.16	F:CAAGGTCCGGGTGCTTACC<br>R:ATTAAAGGGAGGCCCCGAATA
Zic5	0.42	0.33	2.54	F:GCAGCCACGTCTGCTTTTG<br>R:TATGAGTGCGCTTGTGGATCT

Table S4 **Sequences of primers for Foxj3 and Zbtb18**

Primer name	Primer sequence(5′–3′)	Accession Number
Foxj3 -F-NheI	ATTCTGCTAGC**GCCACC**ATGGGTTTGTATGGACAAG	NM_172699.3
Foxj3 -R-BamHI	CGCAGGATCCCACTATTGAATCCCAATCAAAGTC	
Zbtb18-F-BamHI	TTAAGGATCC**GCCACC**ATGTGTCCTAAAGGTTATGAA	NM_001012330.1
Zbtb18-R-BamHI	GCTAGGATCCTTTCCAAAGTTCTTGAGAGCTATCT	

Restriction sites were underlined. Kozak sequence (bold fonts) were included for optimal translation according to manufacturer’s instructions.

Table S5 **Sequences of primers for 3’UTR* of Foxj3 and Zbtb18**

Primer name		Primer sequence(5′–3′)
Foxj3 3’UTR	WT#-F	CCAGGATGACTTTGATTGGGA
	WT-R	AGCCTCTGTATCTTGCTGCCTA
	Site1 mut§ R1	GAATCTCT**TGGCTCA**TTCGAAAGGAATT
	Site1 mut F1	CTAAATTCCTTTCGAA**TGAGCCA**AGAG
	Site2 mut R2	GGACCAATGCTTCT**TGGCTCA**TTTGTCTGT
	Site2 mut F2	CAGACAAA**TGAGCCA**AGAAGCATTGGTCC
Zbtb18 3’UTR	WT-F	GCCAGCTCAGAGTTTAGGT
	WT-R	TTATTGCAGTTAGCACACAGT
	Site1 mut R1	CCACAGACATT**TGGCTCA**TCCTCTAG
	Site1 mut F1	AGGA**TGAGCCA**AATGTCTGTGGAAG
	Site2 mut R2	CATACTAT**TGGCTCA**AAAGCTAGATG
	Site2 mut F2	CTGACATCTAGCTTT**TGAGCCA**ATAGT

Mutations were underlined and showed in bold fonts.

* untranslated region

\# wild type

§ mutation

Table S6 **Sequences of primers for mRNA quantitation**

Gene	Primer name	Sequence (5'—3')	Product length
Foxj3	F	AGCCTAACATCTATGGACTGGT	118 bp
	R	GGTCAAGGAGTGCATTCTTCTTA	
Zbtb18	F	GTTCGGATAAAGTCGAGAGCC	114 bp
	R	CCCTTTTGCTGGGTAGAATGT	
Oct4	F	CGGAAGAGAAAGCGAACTAGC	108 bp
	R	ATTGGCGATGTGAGTGATCTG	
Nestin	F	CCCTGAAGTCGAGGAGCTG	166 bp
	R	CTGCTGCACCTCTAAGCGA	
Map2	F	GCCAGCCTCGGAACAAACA	101 bp
	R	GCTCAGCGAATGAGGAAGGA	
Olig1	F	TCTTCCACCGCATCCCTTCT	226 bp
	R	CCGAGTAGGGTAGGATAACTTC	
Zic5	F	GCAGCCACGTCTGCTTTTG	180 bp
	R	TATGAGTGCGCTTGTGGATCT	
Erbb2	F	ACCGACATGAAGTTGCGACTC	106 bp
	R	AGGTAAGCTCCAAATTGCCCT	
Olig2	F	TCCCCAGAACCCGATGATCTT	90 bp
	R	CGTGGACGAGGACACAGTC	
Actin	F	GGCTGTATTCCCCTCCATCG	154 bp
	R	CCAGTTGGTAACAATGCCATGT	

Table S7 **Sequences of primers for microRNA quantitation***

microRNA	Sequence (5'—3')
217-5P	TACTGCATCAGGAACTGACTGGA
615-5P	GTCCCCGGTGCTCGGAT
216b-5p	GAAATCTCTGCAGGCAAATGTG
615-3p	GAGCCTGGGTCTCCCTCTT
10a-3p	GCAAATTCGTATCTAGGGGAATA
10a-5p	TACCCTGTAGATCCGAATTTGTG
135a-2-3p	TGTAGGGATGGAAGCCATGAA
219-2-3p	AGAATTGTGGCTGGACATCTGT
344d-3p	GGATATAACCACTGCCAGACTGA
99a-5p	AACCCGTAGATCCGATCTTGTG
100-5p	AACCCGTAGATCCGAACTTGTG
1194	GGGAATGAGTAACTGCTAGATCCT
219-5p	TGATTGTCCAAACGCAATTCT
224-5p	GGTAAGTCACTAGTGGTTCCGTT
471-5p	GGGTACGTAGTATAGTGCTTTTCAC
483-5p	AAGACGGGAGAAGAGAAGGGAG
743a-3p	GGAAAGACACCAAGCTGAGTAGA
871-3p	TGACTGGCACCATTCTGGATAAT
470-5p	TTCTTGGACTGGCACTGGTGA
145b	GGTCCAGTTTTCCCAGGAGACT
145a-5p	TCCAGTTTTCCCAGGAATCCCT
134-3p	CTGTGGGCCACCTAGTCACC
296-3p	GAGGGTTGGGTGGAGGCTC

*The reverse primer was 10×miScript Universal Primer provided in miScript II RT Kit (Qiagen)
